# Novel DNA methylation biomarkers in stool and blood for early detection of colorectal cancer and precancerous lesions

**DOI:** 10.1186/s13148-023-01443-7

**Published:** 2023-02-17

**Authors:** Yuguang Shen, Dongyang Wang, Tianli Yuan, Hongsheng Fang, Chen Zhu, Juan Qin, Xiaojing Xu, Cheng Zhang, Jiahua Liu, Yuanruohan Zhang, Zhoujin Wen, Jian Tang, Zheng Wang

**Affiliations:** 1 Department of Gastrointestinal Surgery, Ren Ji Hospital, Shanghai Jiao Tong University School of Medicine, Shanghai, China; 2 Shanghai Biotechnology Corporation, Shanghai, China

**Keywords:** DNA methylation biomarkers, Colorectal cancer, Advanced adenoma, Early detection

## Abstract

**Background:**

Early detection and prevention of precancerous lesions can significantly reduce the morbidity and mortality of colorectal cancer (CRC). Here, we developed new candidate CpG site biomarkers for CRC and evaluated the diagnostic value of their expression in blood and stool samples of CRC and precancerous lesions.

**Methods:**

We analyzed 76 pairs of CRC and adjacent normal tissue samples, 348 stool samples, and 136 blood samples. Candidate biomarkers for CRC were screened using a bioinformatics database and identified using a quantitative methylation-specific PCR method. The methylation levels of the candidate biomarkers were validated using blood and stool samples. The divided stool samples were used to construct and validate a combined diagnostic model and to analyze the independent or combined diagnostic value of candidate biomarkers in stool samples of CRC and precancerous lesions.

**Results:**

Two candidate CpG site biomarkers for CRC, cg13096260 and cg12993163, were identified. Although both biomarkers demonstrated diagnostic performance to a certain extent when using blood samples, they showed better diagnostic value for different stages of CRC and AA with stool samples.

**Conclusions:**

cg13096260 and cg12993163 detection in stool samples could be a promising approach for screening and early diagnosis of CRC and precancerous lesions.

**Supplementary Information:**

The online version contains supplementary material available at 10.1186/s13148-023-01443-7.

## Background

Colorectal cancer (CRC) is an important public health problem, accounting for approximately 10% of global cancer cases and deaths. It is the third most commonly diagnosed cancer and the second leading cause of cancer-related deaths globally [1]. Following the economic growth and westernization of diets in China, both the incidence and mortality of CRC have gradually increased in the recent decade. A similar study indicates that, in China, CRC ranks second and fifth in terms of incidence and mortality, respectively [2]. In contrast, CRC incidence and mortality trends in some developed countries have been steadily declining over time. This change in trend attributes to the country's (like Austria, Germany and France) long-term attention and promotion of CRC screening and early detection, but it is also related to changes in lifestyle and diet [3]. However, the incidence of CRC in young and middle-aged adults is continuously increasing [4].

Most cases of CRC originate from neoplastic precursor lesions over a period of 10–15 years [5]. This time period gives us an opportunity for the screening and surveillance of CRC at an early stage. While the 5-year survival rate of patients with distant stage CRC is 14%, patients with early stage CRC show a better prognosis [4]. Unfortunately, few patients diagnosed with localized disease exhibit symptoms including hematochezia, abdominal pain, or anemia; thus, they often miss the optimal time for diagnosis and treatment. Therefore, it is necessary to enhance CRC secondary prevention.

Currently, the main screening methods for CRC include colonoscopy, flexible sigmoidoscopy, guaiac-based fecal occult blood test (gFOBT), fecal immunochemical test (FIT), stool DNA test, blood-based tests, and computed tomography colonography (CTC) [6]. The effectiveness of CRC screening approaches depends on both patient compliance and the characteristics of the approach being used. Colonoscopy is recognized as the gold standard for preventing CRC; however, sit is expensive and invasive. It also requires intestinal preparation, which results in poor patient compliance. A recent review stated that the risk of perforation for colonoscopy and major bleeding is 4% and 8%, respectively [7]. Although colonoscopy has better sensitivity and specificity of colonoscopy than the majority of screening methods, the miss rate for adenomas is still 26% [8]. These factors limit its popularity. The gFOBT assay has acceptable sensitivity and specificity [9, 10], but it is greatly affected by drugs and diet. The FIT is superior to the gFOBT because it detects human globulin and is therefore unaffected by diet [11]. According to a previous study, FIT has a sensitivity of 79% and a specificity of 94% [12].

Since CRC cells are excreted in feces; abnormal DNA changes during CRC can be detected using fecal samples. Multitarget stool DNA (mt-sDNA) testing is a combination of FIT and aberrant DNA methylation tests. It has a sensitivity of 92.3% for CRC and advanced adenoma (AA) and a specificity of 86.6% [13]. Meanwhile, many studies have shown that the detection of DNA mutations and aberrant methylation in circulating tumor DNA (ctDNA) can provide a new method for CRC screening. DNA methylation can affect tumorigenesis by altering gene expression. CRC cells possess many variable methylation positions [14]. Tumor-specific DNA methylation appears early in the development of tumors [15]. For the early diagnosis of CRC, methods for detecting aberrant methylation of tumor DNA in blood and stool present a novel approach. The US Food and Drug Administration (FDA) has approved a blood-based screening method for CRC that detects methylation of Septin9, a plasma biomarker of CRC, with early CRC sensitivity and specificity of 59% and 79%, respectively [16]. Cologuard, the first stool-based screening method approved by the FDA, includes hemoglobin detection, KRAS gene mutations, and NDRG4 and BMP3 methylation [13]. Despite having high sensitivity and specificity, this method is expensive.

Currently, many candidate DNA methylation biomarkers for CRC have been reported in various studies [17–19]. In one study, a CpG site methylation test of SDC2 in stool samples resulted in a sensitivity of 83–85% for CRC and 48–52% for AA [20]. A combined methylation assay of SDC2 and SEPT9 in stool samples resulted in a sensitivity of 80% for CRC and 57% for AA [21]. SHOX2 has been relatively poorly studied regarding the diagnosis of CRC, but one study has reported that SHOX2 methylation levels were significantly higher in colorectal tumors than in normal tissue [22]. Although some biomarkers show good results, their diagnostic efficacy for early lesions is not strong, thus, limiting their clinical value.

Therefore, this study aimed to explore novel and valuable DNA methylation biomarkers for CRC and precancerous lesions. Candidate biomarkers were identified by bioinformatics analysis. Moreover, we analyzed these candidate biomarkers in clinical tissue, stool, and blood samples to evaluate their clinical performance for the early detection of CRC and precancerous lesions.

## Results

### Discovery of DNA methylation biomarkers and methylation status of candidate CpG sits in The Cancer Genome Atlas (TCGA)

**Fig. 1:**
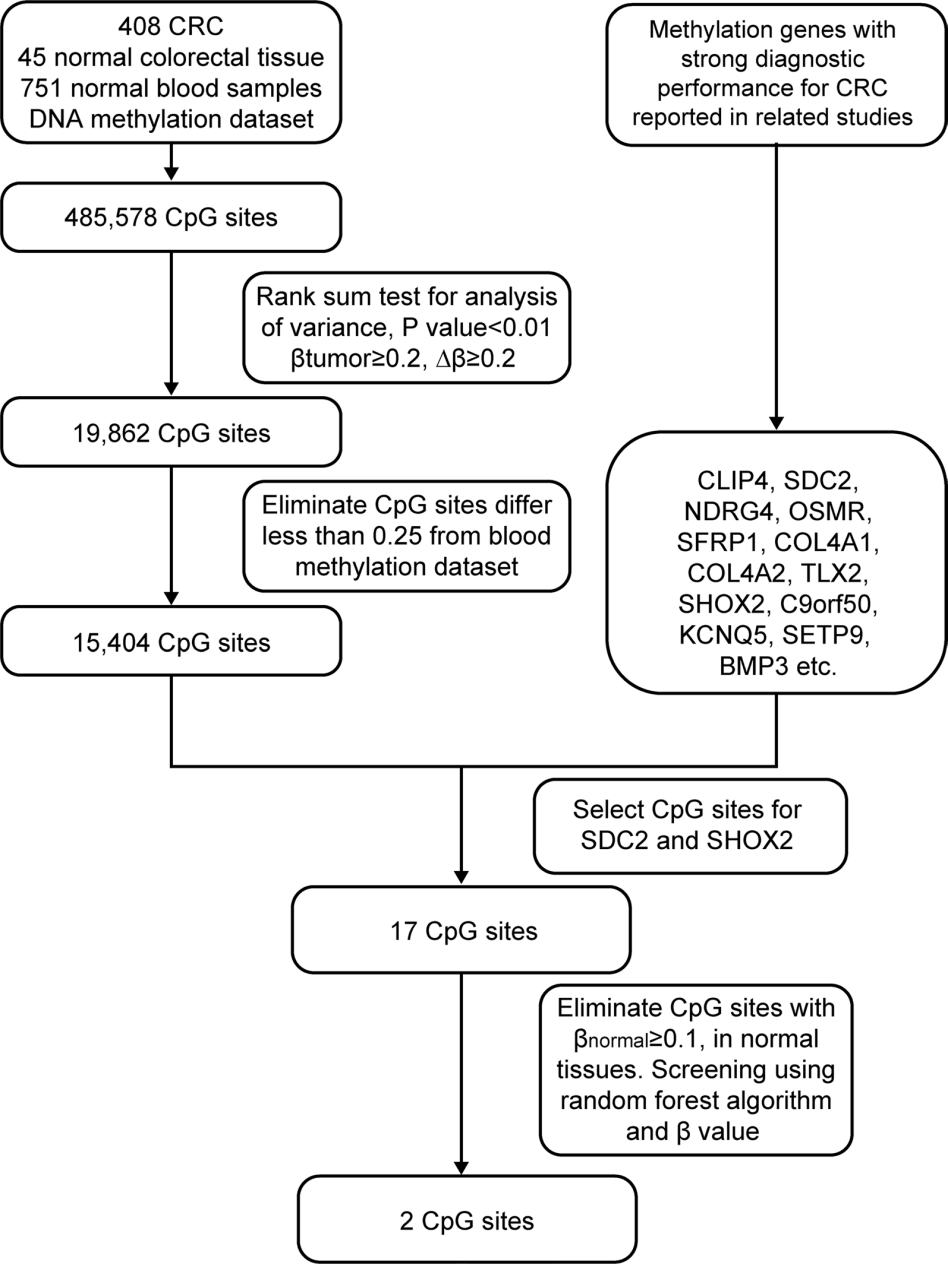
Flowchart of candidate CpG site screening. Initial screening was performed using the DNA methylation dataset of CRC in TCGA. The screening criteria were as follows: the methylation difference between CRC and normal tissues was Δ*β* ≥ 0.2 and was statistically significant (*P* < 0.01), and the methylation value of CpG sites in CRC tissues was *β* ≥ 0.2. Initially, 19,862 CpG sites with differences were screened. Next, the peripheral blood DNA methylation dataset of the non-tumor population in the GEO database was used for rescreening. The methylation levels of CpG sites in CRC tissues were compared with those in blood, and CpG sites with methylation values less than 0.25 were excluded. CpG sites (15,404) were identified. They were also ranked according to differences in their methylation values. Based on the comparison of some of the CRC-related methylation genes, it was found that the CpG sites of SDC2 and SHOX2 genes contained more and ranked higher among the 15,404 methylation sites, with a total of 17 CpG sites. Using the random forest algorithm and selecting CpG sites with low methylation values in normal tissues, the final two candidate CpG sites were obtained. *CRC* colorectal cancer

**Fig. 2:**
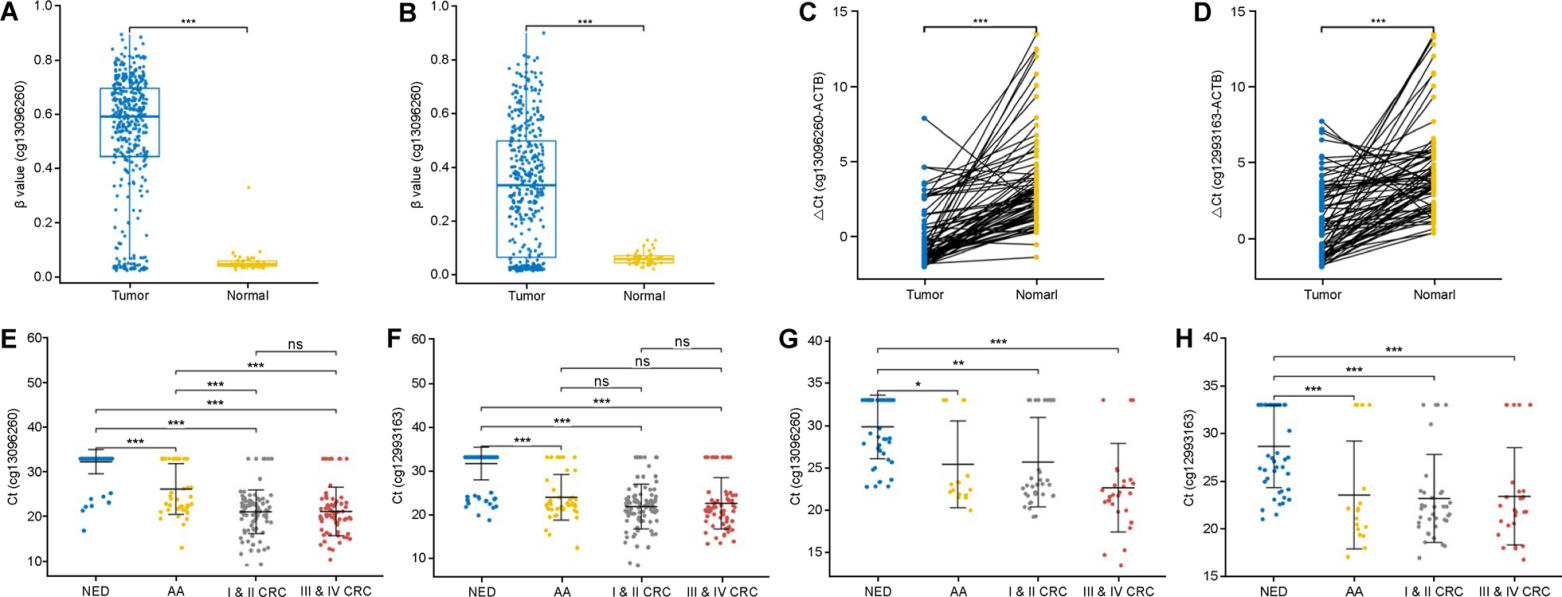
Methylation levels of cg13096260 and cg12993163. Difference analysis of cg13096260 (**a**) and cg12993163 (**b**) in TCGA CRC methylation database. Differential methylation levels of cg13096260 (**c**) and cg12993163 (**d**) in cancerous and normal precancerous tissues of patients with CRC. Methylation levels of cg13096260 (**e**) and cg12993163 (**f**) in stool samples of patients with NED, AA, stage I and II CRC, and stage III&IV CRC. Methylation levels of cg13096260 (**g**) and cg12993163 (**h**) in blood samples of patients with NED, AA, stage I and II CRC, and stage III&IV CRC. *NED* no evidence of disease, *AA* advanced adenoma, *CRC* colorectal cancer

Based on the screening process of CRC-related candidate CpG sites (Fig. 1), and to ensure the diagnostic performance and simplicity of the assay, two candidate CpG sites were selected. cg13096260 was from the *SDC2* promoter region, and cg12993163 was from the of *SHOX2* gene body region. The methylation β values of cg13096260 and cg12993163 were analyzed using TCGA database (Fig. 2A, B). The two candidate CpG sites had significantly higher methylation levels in CRC tissues than in normal tissues (*P* < 0.001).

The relationship between the methylation levels of the two CpG sites and gene expression was analyzed using data from TCGA (Additional file 1: Fig. S1A, B). cg13096260 was negatively correlated with the transcriptional expression of SDC2 (*P* < 0.001), while cg12993163 was not significantly correlated with the transcriptional expression of *SHOX2* (*P* > 0.05). There was a significant positive correlation between the methylation levels of the two CpG sites in CRC tissues (*P* < 0.001) (Additional file 1: Fig. S1C). However, *SDC2* expression was significantly higher in normal tissues (*P* < 0.05), and *SHOX2* expression was significantly higher in tumor tissues (*P* < 0.01) (Additional file 1: Fig. S1D). In addition, survival analysis showed that the lower methylation levels of the two CpG sites corresponded to a better prognosis (*P* < 0.05) (Additional file 1: Fig. S1E, F).

### Biological validation of methylation biomarkers in CRC tissues

To assess the detection limits of quantitative methylation-specific PCR (qMSP) for the two candidate CpG sites and the reference gene, we diluted the synthetic plasmids with candidate CpG site sequences into samples of different concentrations for three replicate assays. Both the candidate CpG sites and the reference gene were detected in samples with a minimum of five copies and a maximum Ct value of 25 (Additional file 1: Table S1). This indicates that the assay is capable of detecting CpG sites in tumor DNA fragments in clinical samples.

To validate the results screened through the database and the methylation levels of candidate CpG sites in CRC tissues, we used qMSP to detect the methylation of cg13096260 and cg12993163 in cancerous and normal precancerous tissues of 76 CRC patients. The ΔCt value was used to indicate the site methylation level, with lower ΔCt values indicating higher methylation levels (Fig. 2C, D). The methylation levels of cg13096260 and cg12993163 were significantly higher in cancer tissues than in normal tissues (*P* < 0.001). The methylation level of cg13096260 was higher in 93.4% (71/76) of tumor tissues than in paired normal tissues, and cg12993163 was higher in 90.8% (69/76) of tumor tissues than in paired normal tissues. This is consistent with the results obtained by analyzing the database.

In addition, we analyzed the relationship between the methylation levels of the two candidate CpG sites and clinicopathological characteristics of the tissue samples. The methylation level of cg12993163 differed among histological types (*P* < 0.05), whereas the rest were not significantly different (Additional file 1: Fig. S2).

### Performance of methylation biomarkers assay on stool samples

The methylation levels of cg13096260 and cg12993163 were detected in 162 CRC, 46 AA, and 120 no evidence of disease (NED) stool samples. Cg13096260 had significantly lower methylation levels in the NED group than in the AA and CRC groups (*P* < 0.001), and the AA group had significantly lower methylation levels than the CRC group (*P* < 0.001) (Fig. 2E, F). There was no significant difference between the early and advanced CRC groups (*P* > 0.05). The methylation level of cg12993163 was significantly lower in the NED group than in the AA and CRC groups (*P* < 0.001), whereas there was no significant difference in methylation levels between the AA and CRC groups (*P* > 0.05).

To construct a combined diagnostic model in stool samples and validate the diagnostic value of the two candidate CpG sites, we randomly divided 328 stool samples into a training set and a validation set in a 2:1 ratio. The training set consisted of stool samples from 108 CRC cases (63 cases of stages I and II and 45 cases of stages III and IV), 31 AA cases, and 80 NED cases. The validation set consisted of stool samples from 54 CRC cases (31 cases of stages I and II, 23 cases of stages III and IV), 15 AA cases, and 40 NED cases. The training set was used to evaluate the predictive ability of two candidate CpG sites for the disease and construct a combined diagnostic model. The validation set was used to establish the cut-off values, determine the clinical significance, and verify the diagnostic efficacy of the two sites.

**Fig. 3:**
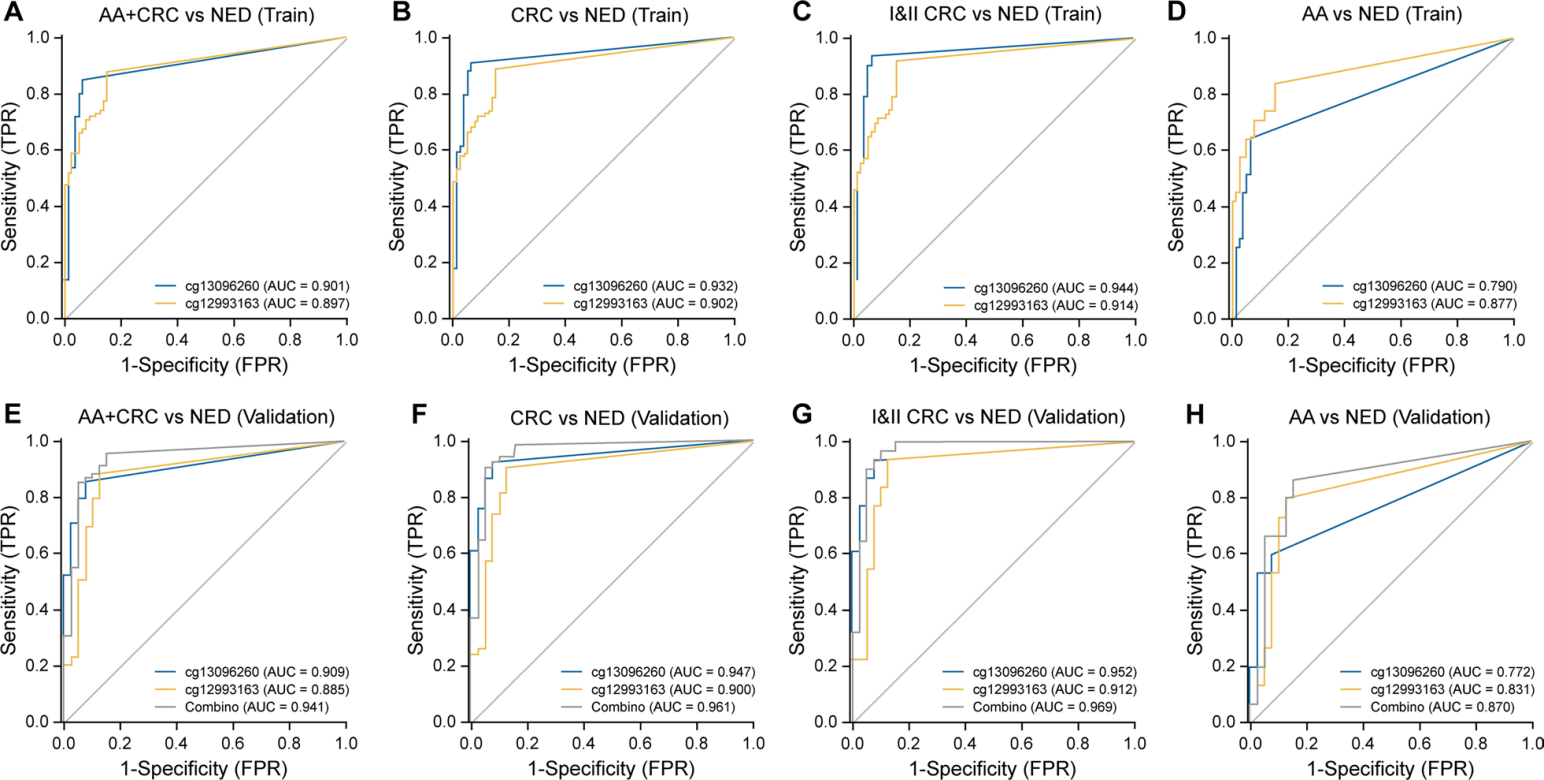
ROC curves and AUC for cg13096260 and cg12993163 in the training set of stool samples. The predictive ability for AA and CRC (**a**), CRC (**b**), I and II CRC (**c**), and AA (**d**). ROC curve and AUC of cg13096260, cg12993163, and the combined diagnostic model in the validation set of stool samples. The predictive ability for AA and CRC (**e**), CRC (**f**), I&II CRC (**g**), and AA (**h**). *AA* advanced adenoma, *CRC* colorectal cancer

Based on the Ct values of the two candidate CpG sites in the training set and their corresponding sample types, ROC curves of the two sites for different grouped samples were obtained, and area under the curve (AUC) values were calculated. The detection results were used to construct a combined logistic diagnostic model (score = 15.7174 − 0.3146 × cg13096260 − 0.2224 × cg12993163) (Fig. 3A–D).

In the validation set, using the Ct values of two candidate CpG sites and diagnostic model scores, ROC curves for different groups were constructed and AUCs were calculated. The AUCs were very close to those in the training set, indicating that two candidate CpG sites had a high discriminatory power for disease (Fig. 3E–H). The AUC of the combined diagnostic model was larger in each group.

**Fig. 4:**
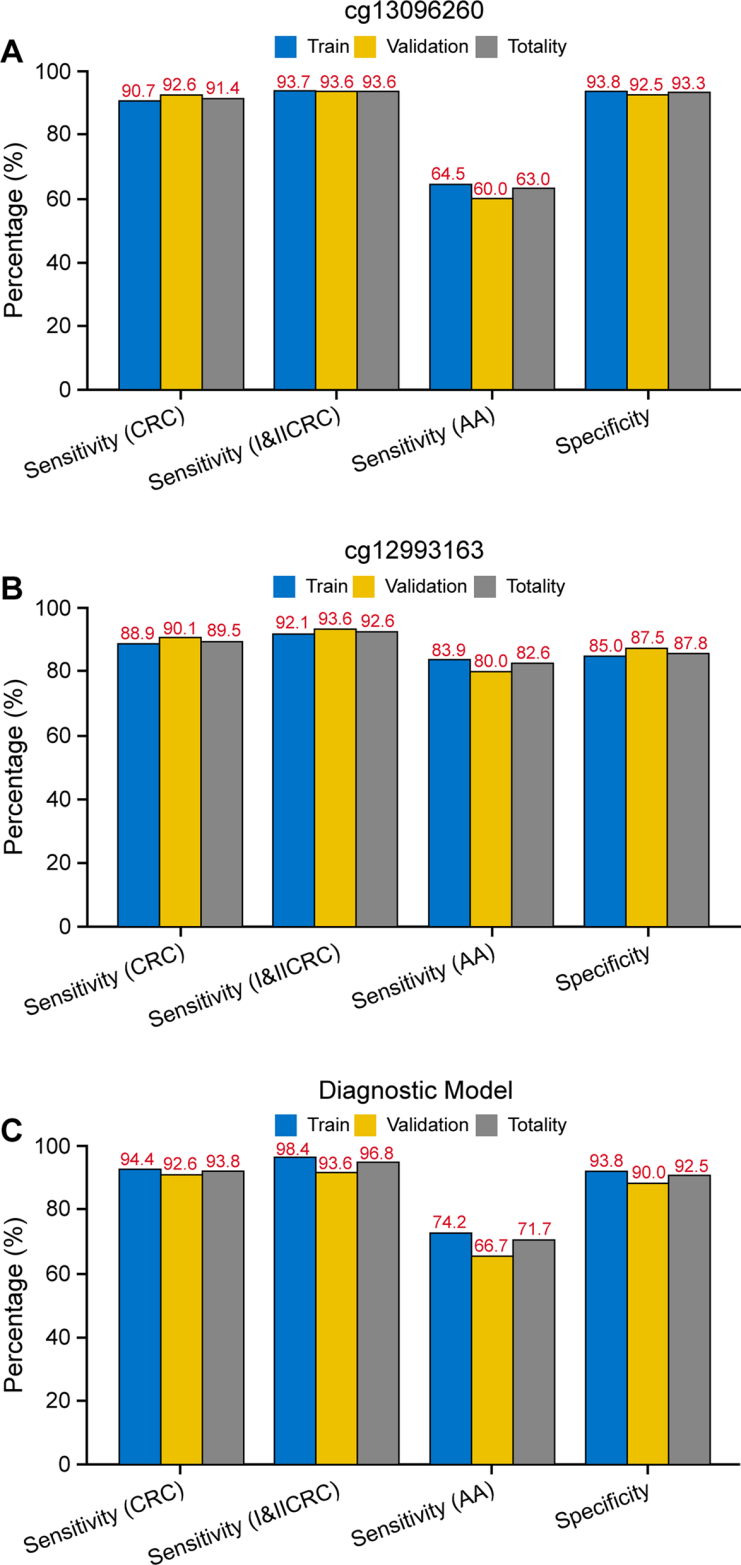
Diagnostic performance in stool samples. Sensitivity and specificity of cg13096260 (**a**), cg12993163 (**b**), and the combined diagnostic model (**c**) for the stool sample training set, validation set, and total samples

The sensitivity and specificity of two candidate CpG sites and the combined diagnostic model for each group of colorectal tumors in different sets were determined (Fig. 4). The sensitivity of cg13096260, cg12993163, and the model for CRC was 91.35% (CI 85.65–95.02%), 89.5% (CI 83.48–93.59%), and 93.83% (CI 88.63–96.83%), respectively. The sensitivity for early CRC was 93.62% (CI 86.09–97.38%), 92.55% (CI 84.75–96.70%), and 96.81% (CI 90.29–99.17%), respectively. The sensitivity for AA was 63.04% (CI 47.53–76.40%), 82.6% (CI 68.05–91.68%), and 71.74% (CI 56.32–83.54%), respectively. The specificities were 93.33% (CI 86.88–96.87%), 85.83% (CI 78.01–91.29%), and 92.5% (CI 85.85–96.30%), respectively.

To further investigate the specificity of DNA methylation detection in stool samples, stool samples from 20 patients with other interfering diseases without colorectal tumors were included in this study and tested for the methylation levels of candidate CpG sites. The negative rates of cg13096260 in other digestive tract tumors and colitis were 81.81% (9/11) and 100% (9/9), respectively, with an overall negative rate of 90%. The negative rates of cg12993163 in other digestive tract tumors and colitis were 72.73% (8/11) and 88.89% (8/9), respectively, with an overall negative rate of 80%. The negative rates of combined diagnostic models for other digestive tract tumors and colitis were 81.81% (9/11) and 100% (9/9), respectively, and the overall negative rate was 90%. The overall negative rate of interfering diseases and the assay specificity of three assays were very similar.

The relationship between stool DNA methylation testing and clinicopathological characteristics of CRC was analyzed. Only cg13096260 and the combined diagnostic model were found to differ among tumor locations (*P* < 0.05), while the rest were not significantly different (Additional file 1: Table S2).

### Performance of methylation marker assays on blood samples

Methylation of two candidate CpG sites was detected in 128 blood samples using qMSP, including 64 CRC, 17 AA, and 47 NED samples. cg13096260 had significantly lower methylation levels in the NED group than in the AA group (*P* < 0.05), stage I and II CRC (*P* < 0.01), and stage III and IV CRC (*P* < 0.001) groups, while there was no significant difference between the AA and CRC group (*P* > 0.05) (Fig. 2G, H). Similarly, cg12993163 had significantly lower methylation levels in the NED group than in the AA and CRC groups (*P* < 0.001), whereas the methylation levels were not significantly different between the AA and CRC groups (*P* > 0.05).

**Fig. 5:**
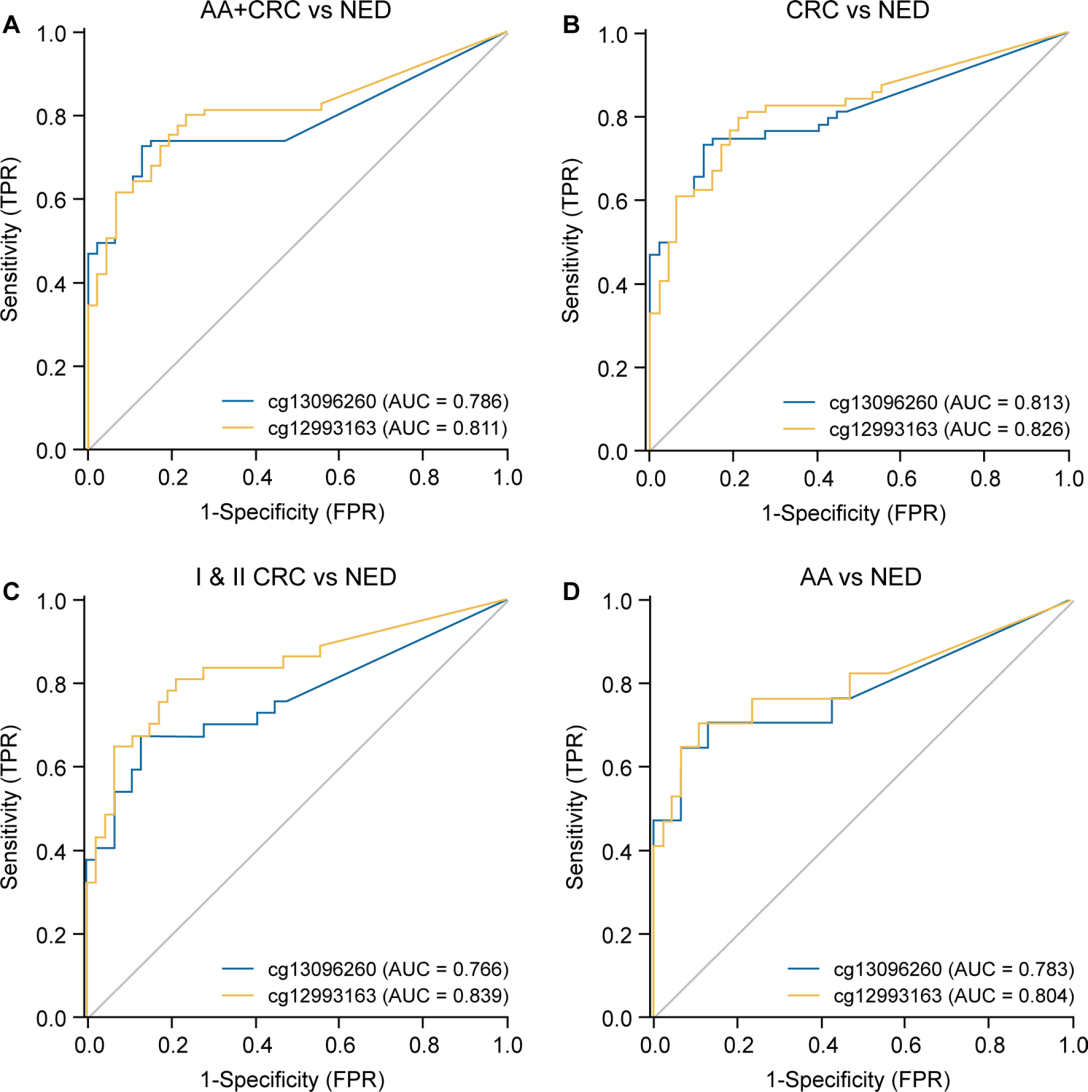
ROC curve and AUC of cg13096260 and cg12993163 in blood samples. The predictive ability for AA and CRC (**a**), CRC (**b**), I and II CRC (**c**), and AA (**d**). *AA* advanced adenoma; *CRC* colorectal cancer

Based on the sample types and their corresponding Ct values, ROC curves for the two sites in different groups were generated, and AUCs were calculated. The results indicated that the diagnostic performance of two candidate CpG sites in blood samples for colorectal tumors was similar (Fig. 5).

The sensitivity of cg13096260 and cg12993163 for CRC was 75% (CI 62.35–84.62%) and 81.25% (CI 69.15–89.53%), respectively; for early CRC was 67.57% (CI 50.11–81.44%) and 81.08% (CI 64.29–91.44%), respectively; and for AA was 70.59% (CI 44.05–88.62%) and 76.47% (CI 49.76–92.18%), respectively. The specificities were 82.98% (CI 68.65–91.86%) and 74.47% (CI 78.01–91.29%), respectively.

To further investigate the specificity of DNA methylation detection in blood samples, blood samples from eight patients with other interfering diseases but without colorectal tumors were included in this study and tested for methylation levels of candidate CpG sites. The negative rate of cg13096260 in both gastric malignancy and colitis was 75% (3/4), and the overall negative rate was 75%. The negative rates of cg12993163 in gastric malignancy and colitis were 50% (2/4) and 75% (3/4), respectively, with an overall negative rate of 62.5%. The overall negative rate of interfering diseases and the assay specificity of two assays were different.

The relationship between blood DNA methylation testing and the clinicopathological characteristics of CRC was analyzed. cg13096260 methylation levels differed based on the presence or absence of vascular invasion (*P* < 0.05). cg12993163 methylation levels differed between male and female participants (*P* < 0.05). The remaining parameters did not differ significantly between the groups (Additional file 1: Table S3).

## Discussion

An important aspect in improving the quality of clinical care and reducing the mortality rate of CRC is the timely detection of precancerous lesions and early CRC [23]. In some developed countries, effective CRC screening tools include gFOBT, FIT, flexible sigmoidoscopy, colonoscopy, CT colonography, and fecal DNA testing [24, 25]. The coverage rate of colonoscopy has exceeded 60%, and more than half of the population aged over 50 years has undergone it. This has largely reduced the incidence and mortality of CRC [4]. It is difficult to fully scale up colonoscopy because it is an invasive test with low adherence and uses up limited health care resources in developing countries.

The most common clinical screening methods for CRC in China include fecal occult blood tests, detection of serum tumor markers, and colonoscopy. Among these, FIT is most commonly used, owing to its higher sensitivity and specificity compared to that of gFOBT; FIT can significantly reduce CRC mortality [26]. However, there remains a risk of missing approximately half of AA and early CRC [27]. The commonly used CRC serum tumor markers include CEA, CA199, and CA125. In our study population, the sensitivities of these three CRC tumor markers were 39.76% (66/166), 24.7% (41/166), and 3.61% (6/166), respectively. The AA detection sensitivities were 9.76% (4/41), 4.88% (2/41), and 2.44% (1/41), respectively. Therefore, the usefulness of serum tumor markers for early CRC screening remains very limited [28]. Only 14% of participants in the CRC screening program underwent colonoscopy, in accordance with the risk factor-based recommendations [29].

To further promote early CRC screening, it is important to develop a simple and convenient blood or stool DNA methylation assay with high performance for early lesion diagnosis. Based on TCGA and Gene Expression Omnibus (GEO) DNA methylation datasets and current research advances, we screened two candidate CpG sites for CRC, cg13096260 and cg12993163. We found that both sites had significantly higher methylation levels in tumor tissues than in normal tissues adjacent to cancer. Cg13096260 is located in the SDC2 promoter region and cg12993163 is located in the SHOX2 gene body region. Both genes are methylation biomarkers for CRC but show limited ability to identify early lesions [22, 30, 31]. We screened and proposed two novel candidate CpG sites which were demonstrated to be more powerful for disease diagnosis.

The common methods currently used to detect DNA methylation include genomic bisulfite sequencing and qMSP [32, 33]. Although high-throughput methylation site detection using genomic bisulfite sequencing is possible, the process is costly, complicated, and time-consuming. Therefore, in this study, we selected qMSP to detect CpG sites. The low cost and simplicity of the assay process make this method suitable for clinical applications and our two-site assay despite its low throughput. We also evaluated the assay’s performance and demonstrated that it could detect methylation of tumor DNA fragments in stool and blood samples.

In this study, we first detected cg13096260 and cg12993163 methylation levels in CRC, AA, and NED stool samples and subsequently constructed and validated a combined diagnostic model using the training and validation sets. The sensitivities of two CpG sites for CRC detection were very similar, whereas cg12993163 was more sensitive for AA detection, and cg13096260 had higher specificity. The combined diagnostic model achieved both high detection specificity and a certain sensitivity for AA. Other interfering diseases had no significant effects on any of the three assays. The clinical diagnostic performance of such a single- or dual-site methylation assay is robust. Moreover, the diagnostic sensitivity of our stool DNA methylation test was not affected by most clinicopathological characteristics, ensuring the same diagnostic sensitivity for different populations and tumor types, which is a required feature for CRC screening and diagnosis.

Stool DNA testing has been used for CRC screening and diagnosis for several years. Cologuard, a stool DNA multi-targeting test kit approved by the US FDA in 2014, has an overall sensitivity of 92.3% for CRC, 42.4% for AA, and a specificity of 86.7% [13]. However, the Cologuard test is expensive, and the test procedure is cumbersome, making it unsuitable for promotion in China. Nevertheless, owing to its high sensitivity for early-stage CRC, it was included in the updated CRC screening guidelines of the National Cancer Society in 2018 [34]. A stool DNA methylation assay was also approved by the Chinese Drug Administration in 2018, which mainly uses PCR to detect the SDC2 methylation levels. The overall sensitivity for CRC was 81.1% and 58.2% for AA, with a 93.3% specificity [30]. In contrast, our assay has higher diagnostic performance than these methods regardless of whether the two sites are used independently or in combination, with lower cost and procedural complexity, making this stool-based assay a viable home screening method for CRC in countries with limited healthcare resources [35]. Thus, it is well suited for replication in developing countries.

Based on the DNA methylation results of blood samples, we found that the diagnostic sensitivity and specificity of cg13096260 and cg12993163 for colorectal tumors were approximately 70–80%, and other interfering diseases may have some influence on the detection of blood samples. Owing to the limited sample size, we did not design the training and validation sets, nor did we evaluate the diagnostic performance after combining the two sites. Although DNA methylation detection in blood samples demonstrated diagnostic performance for colorectal tumors to a certain extent, the diagnostic performance was relatively weak when compared to stool samples. Studies have shown that ctDNA produced by tumor cells in the blood is limited and diluted by whole-body blood, whereas DNA in stool is directly derived from tumor tissue in the intestine. Therefore, tumor DNA in stool is more concentrated and tissue-specific, making CpG sites detection using stool samples more sensitive and specific. This explains our results, which are consistent with those in similar studies [21, 36]. Epi proColon, the first FDA-approved assay based on blood SEPT9 gene methylation, has a sensitivity of 68.2% for CRC and 22% for AA, with a specificity of 78.2% [37]. In another study based on the detection of stool SEPT9 gene methylation, it was found that the methylation level was significantly higher in stool samples than in blood samples. Although both methods perform similarly in diagnosing CRC, the stool test is more sensitive for diagnosing the early stage of the disease than the blood test [36]. Moreover, because of the low sensitivity of Epi proColon for early stage disease, the new National Cancer Society guidelines do not recommend its use for CRC screening [34, 38].

This study has several advantages. First, it is innovative in screening candidate CpG site biomarkers for CRC and validating the CpG sites proposed for the first time in tissue samples. Second, a diverse variety of clinical samples were collected, and rich clinicopathological characteristics were evaluated, ensuring more comprehensive and reliable results. Third, this study provides more objective data to assess the diagnostic value of stool methylation detection through the design of training and validation sets of stool samples. Fourth, the sensitivity of the assays involved in the study was greater than 60% for early stage lesions, and cg12993163 had a sensitivity of 82.6% for AA in stool samples, which provides a significant advantage in both traditional and novel CRC screening and early diagnosis methods.

Nevertheless, this study still has some limitations. First, the study had a limited sample size, especially for AA and blood samples, and some samples were not collected with corresponding clinicopathological characteristics. Second, the excellent detection performance at a single center needs to be validated in a large multicenter population. Third, although other interfering diseases were analyzed, the types of diseases analyzed and number of tests utilized remained very limited. Further expansion of the sample is required for statistical analysis. Fourth, all subjects in this study belonged to a Chinese population with a similar genetic background, and ethnic differences should also be taken into account. Further, the levels of some ctDNAs in blood may follow circadian rhythms [39]. Therefore, the relationship between methylation levels of candidate CpG sites and circadian rhythms in blood and stool samples should be investigated in future studies.

## Conclusions

We developed two candidate CpG site biomarkers for CRC and precancerous lesions: cg13096260 and cg12993163. The two sites were validated to have excellent diagnostic value in stool samples, either independently or in combination, for the detection of CRC at different stages and AA. Thus, detecting cg13096260 and cg12993163 in stool samples may serve as a promising approach for the screening and early diagnosis of CRC and precancerous lesions.

## Methods

### Study design

This study explored and validated novel colorectal tumor biomarkers through the analysis of the biological databases and detection of clinical samples using qMSP. This study focused on the alteration of DNA CpG sites in the stool and blood of patients with colorectal tumor(s) and controls. The clinical trial was registered with the China Clinical Trials Registry, which is a part of the WHO International Clinical Trials Registry, under the trial registration number Chi-CTR-2100048569. The main evaluation indexes include sensitivity and specificity.

### Sample collection

The study enrolled patients who were hospitalized in the Department of Gastrointestinal Surgery of Renji Hospital, Shanghai Jiaotong University School of Medicine between October 2020 and October 2021 and had been pathologically diagnosed with CRC or AA (adenoma ≥ 1 cm in diameter, with high atypical hyperplasia or containing more than 25% of the villi component [40]), as well as some other gastrointestinal diseases (interfering disease). The healthy control group included individuals with NED at the Renji Hospital Medical Examination Center of Shanghai Jiaotong University School of Medicine, who volunteered to participate in the study and underwent endoscopy to exclude the possibility of a digestive tract disease.

Clinical samples collected for this study included cancerous and normal paracancerous tissue samples from patients with CRC, and blood and stool samples from patients with CRC, AA, interfering disease, and NED. Tissue samples were intraoperatively excised from isolated tumor specimens of cancerous tissue and paired with paracancerous normal tissue from the bowel wall tissue. Samples were then snap-frozen in liquid nitrogen and stored at a low temperature of − 80 °C. Stool samples were collected preoperatively from five random locations without bowel preparation, weighing approximately 5 g, and placed in 50 ml centrifuge tubes containing 25 ml of preservation fluid. Long-term storage was performed at − 80 °C. Blood samples were collected preoperatively from approximately 10 ml of whole blood per subject using Streck tubes, separated from the plasma, and stored at − 80 °C for extended periods.

Clinical data collected from the participants included age, sex, tumor size, selected tumor indicators (CEA, CA199, CA125), tumor location, histological type, lymphatic invasion, distant metastasis, pathological staging according to the AJCC 8th edition TNM tumor staging system for CRC, pathological staging, vascular invasion, nerve invasion, microsatellite status, and selected gene mutations (BRAF, PIK3CA, NRAS, and KRAS).

A total of 515 participants were enrolled in this study, and 422 were included in the final analysis. 12 subjects were excluded due to incomplete pathological information, 25 due to unsuccessful collection of tissue, stool, or blood samples, and 32 due to insufficient reference genes. An additional 24 patients with interfering diseases were tested for DNA methylation in stool or blood samples (stool samples included 7 cases of gastric malignancy, 9 cases of colitis, 2 cases of pancreatic malignancy, and 2 cases of liver malignancy; blood samples included 4 cases of gastric malignancy and 4 cases of colitis). Among the 422 participants included in the analysis, 222 had CRC, 46 had AA, and 154 had NED. The flow of sample collection is shown in Additional file 1: Fig. S3. Table 1 lists the main characteristics of each sample, and Additional file 1: Table S4 lists the other characteristics.

**Table 1:** Main characteristics of the study subjects

Group	Characteristics	CRC	AA	NED
Tissue	Number	76		
	Age (year), *n* (%)			
	< 40	0 (0%)		
	40–59	24 (31.6%)		
	60–79	42 (55.3%)		
	≥ 80	10 (13.2%)		
	Mean ± SD	64.8 ± 10.8		
	Gender, *n* (%)			
	Male	44 (57.9%)		
	Female	32 (42.1%)		
Stool	Number	162	46	120
	Age (year), *n* (%)			
	< 40	1 (0.6%)	0 (0%)	14 (11.7%)
	40–59	48 (29.6%)	19 (41.3%)	77 (64.2%)
	60–79	99 (61.1%)	26 (56.5%)	28 (23.3%)
	≥ 80	14 (8.6%)	1 (2.2%)	1 (0.8%)
	Mean ± SD	64.8 ± 10.7	62.8 ± 7.9	51.6 ± 10.3
	Gender, *n* (%)			
	Male	92 (56.8%)	26 (56.5%)	66 (55%)
	Female	70 (43.2%)	20 (43.5%)	54 (45%)
Blood	Number	64	17	47
	Age (year), *n* (%)			
	< 40	1 (1.6%)	0 (0%)	4 (8.5%)
	40–59	21 (32.8%)	7 (41.2%)	16 (34%)
	60–79	33 (51.6%)	10 (58.8%)	25 (53.2%)
	≥ 80	9 (14.1%)	0 (0%)	2 (4.3%)
	Mean ± SD	64.0 ± 12.9	63.5 ± 6.5	61.2 ± 12.7
	Gender, *n* (%)			
	Male	40 (62.5%)	9 (52.9%)	35 (74.5%)
	Female	24 (37.5%)	8 (47.1%)	12 (25.5%)

This study was conducted in accordance with the principles of the Declaration of Helsinki and approved by the Ethics Committee of Renji Hospital, Shanghai Jiaotong University School of Medicine (ethical approval number: KY2021-099-B). All participants signed an informed consent form.

### Biomarker discovery and filter criteria

The public databases involved in this study were TCGA and GEO. Data from TCGA consisted mainly of Infinium Human Methylation450 BeadChip DNA methylation data (Illumina, San Diego, CA, USA), including tumor tissue from 408 CRC patients and 45 normal tissues. In addition, the GSE40279 and GSE41169 peripheral blood DNA methylation datasets from the GEO were downloaded for 751 cases of peripheral blood DNA methylation in non-tumor populations. A stepwise screening approach was used to identify and select CRC-associated DNA methylation biomarker candidates (Fig. 1), resulting in the screening of two candidate CpG sites.

### DNA isolation and methylation testing

Tissue samples were extracted using a DNeasy Blood & Tissue Kit (QIAGEN, 69,581, Düsseldorf, Germany). Stool samples were extracted by grinding the sample with glass beads until completely homogenized and then centrifuged at 4000×*g* for 10 min. The supernatant was collected, and the process was repeated once more, following which the supernatant was again collected. DNA was extracted from supernatant using a MagicPure® Stool and Soil Genomic DNA Kit (Full-Form Gold EC801-11, Beijing, China). DNA from the blood samples was extracted using a MagicPure® Cell-Free DNA Kit (TransGen Biotech, EC201, Beijing, China). All kits were used according to the manufacturers’ instructions, and all collected supernatants were stored at − 20 °C.

EZ DNA Methylation-Gold Kit (ZYMO RESEARCH, D5006, D5006, Los Angeles, CA, USA) was used to transform the extracted DNA samples with sulfite. The assay was performed according to the manufacturer’s instructions. Concentration of the collected DNA was quantified using a spectrophotometer, and the samples were stored at − 20 °C.

Probe Ex Taq (Probe qPCR) (TAKARA, RR390A, Kota Osaka, Japan) kit was used to perform qMSP on DNA from sulfite-transformed samples, and PCR was performed according to the manufacturer’s instructions. Primers and probes were designed according to the specific sequences of CpG sites, with ACTB as the reference gene, refer to Additional file 1: Table S5. Then, 40 μl of the reaction system was configured, containing 10 μl of the DNA template, and three replicate wells were set up for each sample. The samples were spiked in an AB 7500 qPCR instrument (Applied Biosystems, Foster City, CA, USA). A two-step PCR amplification program was set up as follows: pre-denaturation at 95 °C for 30 s, 95 °C for 5 s, and 60 °C for 30 s, repeated for 40 cycles.

### Detection limit of methylation biomarkers assay

Detection limits of the methylation biomarker assay were determined using plasmids with candidate CpG site sequences constructed by Nanjing Kingsray Biotechnology Co. Specific sequences are listed in Additional file 1: Table S6. For qPCR assays, different template concentrations of synthetic plasmids were diluted. A 40 μl reaction system containing 5 μl of DNA template was configured. Three replicate wells were set up for each sample, spiked, and placed in an AB 7500 qPCR instrument. A two-step PCR amplification program was set up as follows: pre-denaturation at 95 °C for 30 s; 95 °C for 5 s, 60 °C for 30 s, repeated for 40 cycles.

### Data analysis

The methylation levels of candidate CpG sites in tissue samples were determined using the ΔCt value which is the difference between the Ct values of the target and reference gene (ACTB) normalized to the amount of DNA in the tissue samples. DNA in the stool and blood samples was mostly fragmented. Therefore, the average Ct value of the three replicate wells obtained using qMSP indicated the detection of the reference gene and the methylation level of the candidate CpG sites. The Ct value of the ACTB reference gene was used to verify the sample quality. If the Ct value of ACTB was greater than 33 in 2/3 of the wells, the sample was considered invalid. If the mean Ct value of cg13096260 in the stool sample was less than 31, it was considered positive for methylation. If the mean Ct value of cg12993163 was less than 32, it was considered positive for methylation. If the mean Ct value of cg13096260 or cg12993163 in the blood samples was less than 25, the samples were considered positive for methylation. If the sample quality was acceptable, but the candidate CpG sites were not detected in 2/3 replicate wells, a Ct value of 33 was assigned to the target CpG sites for subsequent statistical analysis.

When the sample data followed a normal distribution, the *t* test and chi-square test were performed: otherwise, the rank-sum test was performed. The chi-squared test or Fisher's exact probability method was used to compare and correlate groups of clinicopathological data. CpG sites were screened using the rank-sum test and random forest algorithm. The AUC and 95% confidence interval were calculated using the subject operating characteristic curve (ROC curve) to assess the diagnostic performance of candidate CpG sites and guide the cut-off value. A logistic regression model for combined diagnosis was constructed using the glm function. Specificity, sensitivity, and 95% confidence interval were used to assess the clinical value of candidate sites and diagnostic models. All statistical analyses were performed using SPSS 23.0 and R 3.6.3 software. The criteria for determining statistical significance were *P < 0.05, **P < 0.01, ***P < 0.0001, and ns for no statistical significance.

## Supplementary Information

**Additional file 1: Table S1.** Limit of detection of methylation markers. **Table S2**. Relationship between the sensitivity of methylation biomarker assays in stool samples of CRC and clinicopathological characteristics. **Table S3**. Relationship between sensitivities of methylation biomarkers assay in blood samples of CRC and clinicopathological characteristics. **Table S4**. Characteristics of the study subjects. **Table S5**. Primer and probe sequences for CpG sites and reference gene. **Table S6**. Artificially synthesized plasmid sequences. **Figure S1**. Relationship between CpG site methylation levels and gene expression according to data from TCGA. Correlation between cg13096260 methylation levels and SDC2 gene transcript expression (**a**). Correlation between cg12993163 methylation levels and SHOX2 gene transcript expression (**b**). Correlation between cg13096260 methylation levels and cg12993163 methylation levels (**c**). Differences in the expression of SDC2 and SHOX2 genes between tumor and normal tissues (**d**). Survival analysis of cg13096260 methylation levels in colorectal cancer (**e**). Survival analysis of cg13096260 methylation levels in colorectal cancer (**f**). **Figure S2**. Relationship between methylation levels of cg13096260 and cg12993163, and clinicopathological characteristics of tissue samples. **a**–**i** show age, sex, tumor size, tumor location, histological type, lymphatic invasion, distant metastasis, PIK3CA mutation, and KRAS mutation. **Figure S3**. Flowchart of sample collection.

## Data Availability

The datasets used and/or analyzed during the current study are available from the corresponding author on reasonable request.

## Abbreviations

TCGA: The Cancer Genome Atlas
GEO: Gene expression omnibus
qMSP: Quantitative real-time methylation specific PCR
ROC: Receiver operating characteristic
AUC: Area under curve
AA: Advanced adenoma
CRC: Colorectal cancer
NED: No evidence of disease
FIT: Fecal immunochemical test
gFOBT: Guaiac fecal occult blood test
