# Supplementary material for: Novel DNA methylation biomarkers in stool and blood for early detection of colorectal cancer and precancerous lesions
Source: Clin Epigenetics. 2023 Feb 17;15:26. doi: 10.1186/s13148-023-01443-7 (PMC9938553; doi:10.1186/s13148-023-01443-7)
Supplement: Supplementary file 1 — Additional file 1: Table S1. Limit of detection of methylation markers. Table S2. Relationship between the sensitivity of methylation biomarker assays in stool samples of CRC and clinicopathological characteristics. Table S3. Relationship between sensitivities of methylation biomarkers assay in blood samples of CRC and clinicopathological characteristics. Table S4. Characteristics of the study subjects. Table S5. Primer and probe sequences for CpG sites and reference gene. Table S6. Artificially synthesized plasmid sequences. Figure S1. Relationship between CpG site methylation levels and gene expression according to data from TCGA. Correlation between cg13096260 methylation levels and SDC2 gene transcript expression (a). Correlation between cg12993163 methylation levels and SHOX2 gene transcript expression (b). Correlation between cg13096260 methylation levels and cg12993163 methylation levels (c). Differences in the expression of SDC2 and SHOX2 genes between tumor and normal tissues (d). Survival analysis of cg13096260 methylation levels in colorectal cancer (e). Survival analysis of cg13096260 methylation levels in colorectal cancer (f). Figure S2. Relationship between methylation levels of cg13096260 and cg12993163, and clinicopathological characteristics of tissue samples. a–i show age, sex, tumor size, tumor location, histological type, lymphatic invasion, distant metastasis, PIK3CA mutation, and KRAS mutation. Figure S3. Flowchart of sample collection. [file 13148_2023_1443_MOESM1_ESM.docx]

**Additional file**

**Additional file 1: Table S1**. Limit of detection of methylation markers.

| Copies | ACTB | cg13096260 | cg12993163 | Detection rate |
| --- | --- | --- | --- | --- |
| 1×10^4^ | 13.21 | 12.27 | 11.95 | 100% (3/3) |
| 1×10^3^ | 16.21 | 15.57 | 15.23 | 100% (3/3) |
| 1×10^2^ | 19.38 | 19.08 | 18.42 | 100% (3/3) |
| 50 | 19.52 | 19.46 | 18.91 | 100% (3/3) |
| 25 | 20.65 | 19.98 | 19.71 | 100% (3/3) |
| 10 | 21.74 | 21.52 | 20.88 | 100% (3/3) |
| 5 | 22.28 | 24.95 | 21.32 | 100% (3/3) |
| 0 | Undetermined | Undetermined | Undetermined | 0% (0/3) |

The numbers in the table represent the average Ct values of the three qPCR assays.

**Additional file 1: Table S2**. Relationship between the sensitivity of methylation biomarker assays in stool samples of CRC and clinicopathological characteristics.

| Characteristics | cg13096260 | | cg12993163 | | Diagnostic model | |
| --- | --- | --- | --- | --- | --- | --- |
|  | Sensitivity (%) | P value | Sensitivity (%) | P value | Sensitivity (%) | P value |
| Age (year) |  |  |  |  |  |  |
| <40 | 100 | 0.662 | 100 | 0.752 | 100 | 0.545 |
| 40–59 | 89.6 |  | 91.7 |  | 95.8 |  |
| 60–79 | 90.9 |  | 88.9 |  | 91.9 |  |
| ≥80 | 100 |  | 85.7 |  | 100 |  |
| Gender |  |  |  |  |  |  |
| Male | 91.3 | 1.000 | 88 | 0.662 | 92.4 | 0.517 |
| Female | 91.4 |  | 91.4 |  | 95.7 |  |
| Size (cm) |  |  |  |  |  |  |
| 1–2 | 100 | 0.248 | 100 | 0.442 | 100 | 0.077 |
| 2–4 | 87.9 |  | 86.8 |  | 90.1 |  |
| >4 | 95.7 |  | 92.8 |  | 98.6 |  |
| CEA (ng/ml) |  |  |  |  |  |  |
| ≤5 | 89.6 | 0.162 | 89.6 | 0.549 | 92.7 | 0.189 |
| >5 | 95.2 |  | 90.3 |  | 96.8 |  |
| N/A | 75 |  | 75 |  | 75 |  |
| CA199 (U/ml) |  |  |  |  |  |  |
| ≤27 | 93.3 | 0.149 | 90.8 | 0.302 | 95 | 0.205 |
| >27 | 86.8 |  | 86.8 |  | 92.1 |  |
| N/A | 75 |  | 75 |  | 75 |  |
| CA125 (U/ml) |  |  |  |  |  |  |
| ≤35 | 91.4 | 0.368 | 89.5 | 0.442 | 94.1 | 0.263 |
| >35 | 100 |  | 100 |  | 100 |  |
| N/A | 75 |  | 75 |  | 75 |  |
| Location |  |  |  |  |  |  |
| Left | 93.9 | 0.011 | 91.8 | 0.486 | 95.9 | 0.029 |
| Right | 78.9 |  | 84.2 |  | 84.2 |  |
| Rectum | 96 |  | 90.7 |  | 97.3 |  |
| Histological type |  |  |  |  |  |  |
| Adenocarcinoma | 91.1 | 0.122 | 89.7 | 0.717 | 93.8 | 1.000 |
| Mucinous | 100 |  | 85.7 |  | 92.9 |  |
| Signet ring cell | 50 |  | 100 |  | 100 |  |
| Lymphatic invasion |  |  |  |  |  |  |
| Positive | 88.2 | 0.358 | 85.3 | 0.219 | 89.7 | 0.096 |
| Negative | 93.6 |  | 92.6 |  | 96.8 |  |
| Distant metastasis |  |  |  |  |  |  |
| Positive | 76.9 | 0.088 | 76.9 | 0.141 | 84.6 | 0.185 |
| Negative | 92.6 |  | 90.6 |  | 94.6 |  |
| Stage |  |  |  |  |  |  |
| Ⅰ | 92.3 | 0.242 | 96.2 | 0.247 | 100 | 0.151 |
| Ⅱ | 94.1 |  | 91.2 |  | 95.6 |  |
| Ⅲ | 90.9 |  | 87.3 |  | 90.9 |  |
| Ⅳ | 76.9 |  | 76.9 |  | 84.6 |  |
| Vascular invasion |  |  |  |  |  |  |
| Positive | 91.9 | 0.926 | 85.1 | 0.062 | 91.9 | 0.803 |
| Negative | 91.3 |  | 95.7 |  | 95.7 |  |
| N/A | 89.5 |  | 84.2 |  | 94.7 |  |
| Nerve invasion |  |  |  |  |  |  |
| Positive | 91.2 | 1.000 | 86.8 | 0.288 | 91.2 | 0.402 |
| Negative | 91.1 |  | 95.6 |  | 97.8 |  |
| N/A | 92.3 |  | 88.5 |  | 96.2 |  |
| Microsatellite status |  |  |  |  |  |  |
| MSS | 90.1 | 0.560 | 90.8 | 0.057 | 92.9 | 0.692 |
| MSI | 100 |  | 50 |  | 100 |  |
| N/A | 100 |  | 88.2 |  | 100 |  |
| BRAF mutation |  |  |  |  |  |  |
| Positive | 85.7 | 0.245 | 100 | 1.000 | 100 | 0.594 |
| Negative | 90.3 |  | 88.8 |  | 92.5 |  |
| N/A | 100 |  | 90.5 |  | 100 |  |
| PIK3CA mutation |  |  |  |  |  |  |
| Positive | 100 | 0.316 | 80 | 0.572 | 100 | 0.537 |
| Negative | 89.7 |  | 89.7 |  | 92.6 |  |
| N/A | 100 |  | 90.5 |  | 100 |  |
| NRAS mutation |  |  |  |  |  |  |
| Positive | 100 | 0.405 | 100 | 1.000 | 100 | 0.473 |
| Negative | 89.9 |  | 89.1 |  | 92.8 |  |
| N/A | 100 |  | 90.5 |  | 100 |  |
| KRAS mutation |  |  |  |  |  |  |
| Positive | 88.3 | 0.260 | 95 | 0.175 | 100 | 0.536 |
| Negative | 91.4 |  | 85.2 |  | 93.8 |  |
| N/A | 100 |  | 90.5 |  | 91.7 |  |

**Additional file 1: Table S3**. Relationship between sensitivities of methylation biomarkers assay in blood samples of CRC and clinicopathological characteristics.

| Characteristics | cg13096260 | | cg12993163 | |
| --- | --- | --- | --- | --- |
|  | Sensitivity (%) | P value | Sensitivity (%) | P value |
| Age (year) |  |  |  |  |
| <40 | 100 | 0.183 | 100 | 0.927 |
| 40–59 | 76.2 |  | 81 |  |
| 60–79 | 66.7 |  | 78.8 |  |
| ≥80 | 100 |  | 88.9 |  |
| Gender |  |  |  |  |
| Male | 67.5 | 0.136 | 72.5 | 0.023 |
| Female | 87.5 |  | 95.8 |  |
| Size (cm) |  |  |  |  |
| 1–2 | 66.7 | 0.522 | 83.3 | 0.795 |
| 2–4 | 70 |  | 76.7 |  |
| >4 | 82.1 |  | 85.7 |  |
| CEA (ng/ml) |  |  |  |  |
| ≤5 | 73.7 | 1.000 | 76.3 | 0.331 |
| >5 | 76.9 |  | 88.5 |  |
| CA199 (U/ml) |  |  |  |  |
| ≤27 | 75.6 | 1.000 | 77.8 | 0.484 |
| >27 | 73.7 |  | 89.5 |  |
| CA125 (U/ml) |  |  |  |  |
| ≤35 | 75.8 | 0.440 | 80.6 | 1.000 |
| >35 | 50 |  | 100 |  |
| Location |  |  |  |  |
| Left | 73.7 | 0.695 | 73.7 | 0.347 |
| Right | 85.7 |  | 92.9 |  |
| Rectum | 71 |  | 80.6 |  |
| Histological type |  |  |  |  |
| Adenocarcinoma | 73.7 | 1.000 | 82.5 | 0.448 |
| Mucinous | 83.3 |  | 66.7 |  |
| Signet ring cell | 100 |  | 100 |  |
| Lymphatic invasion |  |  |  |  |
| Positive | 84.6 | 0.240 | 80.8 | 1.000 |
| Negative | 68.4 |  | 81.6 |  |
| Distant metastasis |  |  |  |  |
| Positive | 100 | 0.319 | 100 | 0.574 |
| Negative | 72.9 |  | 79.7 |  |
| Stage |  |  |  |  |
| Ⅰ | 66.7 | 0.418 | 75 | 0.712 |
| Ⅱ | 68 |  | 84 |  |
| Ⅲ | 81.8 |  | 77.3 |  |
| Ⅳ | 100 |  | 100 |  |
| Vascular invasion |  |  |  |  |
| Positive | 90 | 0.026 | 83.3 | 0.573 |
| Negative | 62.5 |  | 83.3 |  |
| N/A | 60 |  | 70 |  |
| Nerve invasion |  |  |  |  |
| Positive | 85.3 | 0.113 | 88.2 | 0.268 |
| Negative | 64.7 |  | 70.6 |  |
| N/A | 61.5 |  | 76.9 |  |
| Microsatellite status |  |  |  |  |
| MSS | 75 | 0.800 | 82.1 | 0.558 |
| MSI | 100 |  | 100 |  |
| N/A | 66.7 |  | 66.7 |  |
| BRAF mutation |  |  |  |  |
| Positive | 100 | 0.718 | 100 | 0.671 |
| Negative | 73.6 |  | 81.1 |  |
| N/A | 71.4 |  | 71.4 |  |
| PIK3CA mutation |  |  |  |  |
| Positive | 100 | 0.857 | 100 | 0.796 |
| Negative | 74.1 |  | 81.5 |  |
| N/A | 71.4 |  | 71.4 |  |
| NRAS mutation |  |  |  |  |
| Positive | 100 | 1.000 | 100 | 0.745 |
| Negative | 74.5 |  | 81.8 |  |
| N/A | 71.4 |  | 71.4 |  |
| KRAS mutation |  |  |  |  |
| Positive | 73.1 | 0.922 | 88.5 | 0.393 |
| Negative | 77.4 |  | 77.4 |  |
| N/A | 71.4 |  | 71.4 |  |

**Additional file 1: Table S4**. Characteristics of the study subjects.

| Characteristics | Tissue |  | Stool | | |  | Blood | | |
| --- | --- | --- | --- | --- | --- | --- | --- | --- | --- |
|  | CRC |  | CRC | AA | NED |  | CRC | AA | NED |
| Number | 76 |  | 162 | 46 | 120 |  | 64 | 17 | 47 |
| Age (year) |  |  |  |  |  |  |  |  |  |
| <40 | 0 (0%) |  | 1 (0.6%) | 0 (0%) | 14 (11.7%) |  | 1 (1.6%) | 0 (0%) | 4 (8.5%) |
| 40–59 | 24 (31.6%) |  | 48 (29.6%) | 19 (41.3%) | 77 (64.2%) |  | 21 (32.8%) | 7 (41.2%) | 16 (34%) |
| 60–79 | 42 (55.3%) |  | 99 (61.1%) | 26 (56.5%) | 28 (23.3%) |  | 33 (51.6%) | 10 (58.8%) | 25 (53.2%) |
| ≥80 | 10 (13.2%) |  | 14 (8.6%) | 1 (2.2%) | 1 (0.8%) |  | 9 (14.1%) | 0 (0%) | 2 (4.3%) |
| mean ± SD | 64.8 ± 10.8 |  | 64.8 ± 10.7 | 62.8 ± 7.9 | 51.6 ± 10.3 |  | 64.0 ± 12.9 | 63.5 ± 6.5 | 61.2 ± 12.7 |
| Gender |  |  |  |  |  |  |  |  |  |
| Male | 44 (57.9%) |  | 92 (56.8%) | 26 (56.5%) | 66 (55%) |  | 40 (62.5%) | 9 (52.9%) | 35 (74.5%) |
| Female | 32 (42.1%) |  | 70 (43.2%) | 20 (43.5%) | 54 (45%) |  | 24 (37.5%) | 8 (47.1%) | 12 (25.5%) |
| Size (cm) |  |  |  |  |  |  |  |  |  |
| 1–2 | 44 (57.9%) |  | 2 (1.2%) | 22 (47.8%) |  |  | 6 (9.4%) | 14 (82.4%) |  |
| 2–4 | 32 (42.1%) |  | 91(56.2%) | 24 (52.2%) |  |  | 30 (46.9%) | 3 (17.6%) |  |
| >4 | 76 |  | 69 (42.6%) |  |  |  | 28 (43.8%) | 0 (0%) |  |
| CEA (ng/ml) |  |  |  |  |  |  |  |  |  |
| ≤5 |  |  | 96 (59.3%) | 37 (80.4%) |  |  | 38 (59.4%) | 11 (64.7%) |  |
| >5 |  |  | 62 (38.3%) | 4 (8.7%) |  |  | 26 (40.6%) | 3 (17.6%) |  |
| N/A |  |  | 4 (2.5%) | 5 (10.9%) |  |  | 0 (0%) | 3 (17.6%) |  |
| CA199 (U/ml) |  |  |  |  |  |  |  |  |  |
| ≤27 |  |  | 120 (74.1%) | 39 (84.8%) |  |  | 45 (70.3%) | 14 (82.4%) |  |
| >27 |  |  | 38 (23.5%) | 2 (4.3%) |  |  | 19 (29.7%) | 0 (0%) |  |
| N/A |  |  | 4 (2.5%) | 5 (10.9%) |  |  | 0 (0%) | 3 (17.6%) |  |
| CA125 (U/ml) |  |  |  |  |  |  |  |  |  |
| ≤35 |  |  | 152 (93.8%) | 40 (87%) |  |  | 62 (96.9%) | 14 (82.4%) |  |
| >35 |  |  | 6 (3.7%) | 1 (2.2%) |  |  | 2 (3.1%) | 0 (0%) |  |
| N/A |  |  | 4 (2.5%) | 5 (10.9%) |  |  | 0 (0%) | 3 (17.6%) |  |
| Location |  |  |  |  |  |  |  |  |  |
| Left | 22 (28.9%) |  | 49 (30.2%) |  |  |  | 19 (29.7%) |  |  |
| Right | 21 (27.6%) |  | 38 (23.5%) |  |  |  | 14 (21.9%) |  |  |
| Rectum | 33 (43.4%) |  | 75 (46.3%) |  |  |  | 31 (48.4%) |  |  |
| Histological type |  |  |  |  |  |  |  |  |  |
| Adenocarcinoma | 65 (85.5%) |  | 146 (90.1%) |  |  |  | 57 (89.1%) |  |  |
| Mucinous | 11 (14.5%) |  | 14 (8.6%) |  |  |  | 6 (9.4%) |  |  |
| Signet ring cell | 0 (0%) |  | 2 (1.2%) |  |  |  | 1 (1.6%) |  |  |
| Lymphatic invasion |  |  |  |  |  |  |  |  |  |
| Positive | 51 (67.1%) |  | 68 (42%) |  |  |  | 26 (40.6%) |  |  |
| Negative | 25 (32.9%) |  | 94 (58%) |  |  |  | 38 (59.4%) |  |  |
| Distant metastasis |  |  |  |  |  |  |  |  |  |
| Positive | 70 (92.1%) |  | 13 (8%) |  |  |  | 5 (7.8%) |  |  |
| Negative | 6 (7.9%) |  | 149 (92%) |  |  |  | 59 (92.2%) |  |  |
| Stage |  |  |  |  |  |  |  |  |  |
| Ⅰ | 10 (13.2%) |  | 26 (16%) |  |  |  | 12 (18.8%) |  |  |
| Ⅱ | 40 (52.6%) |  | 68 (42%) |  |  |  | 25 (39.1%) |  |  |
| Ⅲ | 20 (26.3%) |  | 55 (34%) |  |  |  | 22 (34.4%) |  |  |
| Ⅳ | 6 (7.9%) |  | 13 (8%) |  |  |  | 5 (7.8%) |  |  |
| Vascular invasion |  |  |  |  |  |  |  |  |  |
| Positive |  |  | 74 (45.7%) |  |  |  | 30 (46.9%) |  |  |
| Negative |  |  | 69 (42.6%) |  |  |  | 24 (37.5%) |  |  |
| N/A |  |  | 19 (11.7%) |  |  |  | 10 (15.6%) |  |  |
| Nerve invasion |  |  |  |  |  |  |  |  |  |
| Positive |  |  | 91 (56.2%) |  |  |  | 34 (53.1%) |  |  |
| Negative |  |  | 45 (27.8%) |  |  |  | 17 (26.6%) |  |  |
| N/A |  |  | 26 (16%) |  |  |  | 13 (20.3%) |  |  |
| Microsatellite status |  |  |  |  |  |  |  |  |  |
| MSS | 37 (48.7%) |  | 141 (87%) |  |  |  | 56 (87.5%) |  |  |
| MSI | 1 (1.3%) |  | 4 (2.5%) |  |  |  | 2 (3.1%) |  |  |
| N/A | 38 (50%) |  | 17 (10.5%) |  |  |  | 6 (9.4%) |  |  |
| BRAF mutation |  |  |  |  |  |  |  |  |  |
| Positive | 0 (0%) |  | 7 (4.3%) |  |  |  | 4 (6.2%) |  |  |
| Negative | 37 (48.7%) |  | 134 (82.7%) |  |  |  | 53 (82.8%) |  |  |
| N/A | 39 (51.3%) |  | 21 (13%) |  |  |  | 7 (10.9%) |  |  |
| PIK3CA mutation |  |  |  |  |  |  |  |  |  |
| Positive | 3 (3.9%) |  | 5 (3.1%) |  |  |  | 3 (4.7%) |  |  |
| Negative | 34 (44.7%) |  | 136 (84%) |  |  |  | 54 (84.4%) |  |  |
| N/A | 39 (51.3%) |  | 21 (13%) |  |  |  | 7 (10.9%) |  |  |
| NRAS mutation |  |  |  |  |  |  |  |  |  |
| Positive | 1 (1.3%) |  | 3 (1.9%) |  |  |  | 2 (3.1%) |  |  |
| Negative | 40 (52.6%) |  | 138 (85.2%) |  |  |  | 55 (85.9%) |  |  |
| N/A | 35 (46.1%) |  | 21 (13%) |  |  |  | 7 (10.9%) |  |  |
| KRAS mutation |  |  |  |  |  |  |  |  |  |
| Positive | 29 (38.2%) |  | 60 (37%) |  |  |  | 26 (40.6%) |  |  |
| Negative | 24 (31.6%) |  | 81 (50%) |  |  |  | 31 (48.4%) |  |  |
| N/A | 23 (30.3%) |  | 21 (13%) |  |  |  | 7 (10.9%) |  |  |

**Additional file 1: Table S5**. Primer and probe sequences for CpG sites and reference gene.

| Primer/probe | Sequence (5′-3′) |
| --- | --- |
| cg13096260-forward | GTAGGAGTTTTGGTTTGTCGGTG |
| cg13096260-reverse | ATTCCTACAAAATTACACGCCGA |
| cg13096260-probe | AGAGTCGGCGTAGTTAT |
| cg12993163-forward | CGTTTCGTTTGTTCGATCG |
| cg12993163-reverse | CTAACCCGACTTAAACGACGA |
| cg12993163-probe | TACGAGTATAGGCGTTTAC |
| ACTB-forward | GGTGATGGAGGAGGTTTAGTAAGTTT |
| ACTB-reverse | CACCACCCAACACACAATAACAA |
| ACTB-probe | TGGATTGTGAATTTGTG |

**Additional file 1: Table S6.** Artificially synthesized plasmid sequences.

| CpG site | Sequence (5′-3′) |
| --- | --- |
| cg13096260 | ATTAATTGTTTTTTAGAAAAGGGAAAGTGAAGAAGGGAAAGAGAAAAGATAACGGGGAAGAAAAGAGTATAGAGGAGAGAGGAAAAGTGGGGAGAGAAAGGAAGAAAAGGATTGAGAAAACGTAGGAGTTTTGGTTTGTCGGTGAGTAGAGTCGGCGTAGTTATAGCGCGGAGTCGCGGCGTTTATTGGTTTTCGGAGTTGTTAATCGGCGTGTAATTTTGTAGGAATTTTTTTCGGGTTTATTTGGGAGTTATATTGTCGTTTTTTTTTTTTAGTCGTTTAGGGGAGTTCGGAGAAGTAGGTTTAGGAGGGAGGGAGTTAGAGGAAAAGAAGAGGAGGAGAAGGAGGA |
| cg12993163 | TCGGTGTTGTGTCGTATAGGGAGTCGTATTCGTAGACGTTTTTCGTTGTTTTTGGGTTCGGGTTAAATTTTGTATAAGGTTTTTTGGATAGTTAGGTAATTTTCGTTTCGTTTGTTCGATCGGGGTCGTACGAGTATAGGCGTTTACGTTATGTTGGTTGTTTAAAGGGTTCGTCGTTTAAGTCGGGTTAGAAGGTAGGAGGCGGAAAATTAGTTTTCGGTGGCGGGCGAAAGTAATCGTTTTTTTTGTTTTTTTTTCGTTTTTTTTCGTGGAAACGTAGATTCGATTTTAAACGTTTAA |


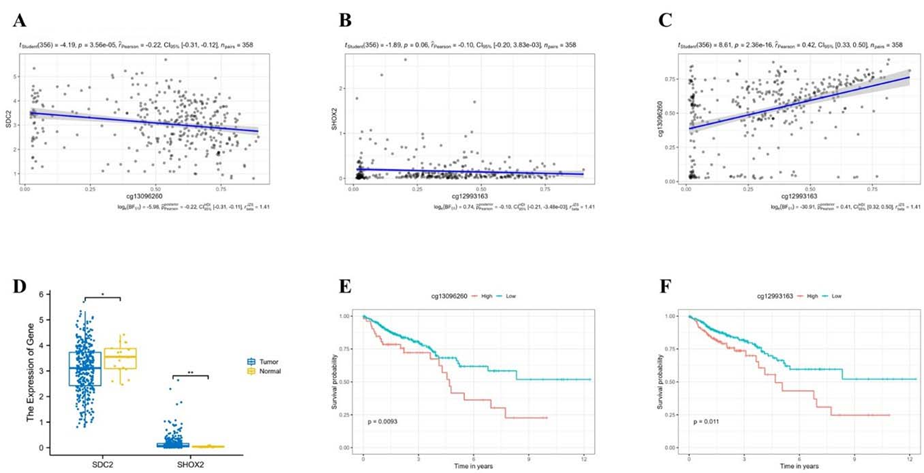


**Additional file 1: Figure S1**. Relationship between CpG site methylation levels and gene expression according to data from TCGA. Correlation between cg13096260 methylation levels and SDC2 gene transcript expression (**a**). Correlation between cg12993163 methylation levels and SHOX2 gene transcript expression (**b**). Correlation between cg13096260 methylation levels and cg12993163 methylation levels (**c**). Differences in the expression of SDC2 and SHOX2 genes between tumor and normal tissues (**d**). Survival analysis of cg13096260 methylation levels in colorectal cancer (**e**). Survival analysis of cg13096260 methylation levels in colorectal cancer (**f**).

**
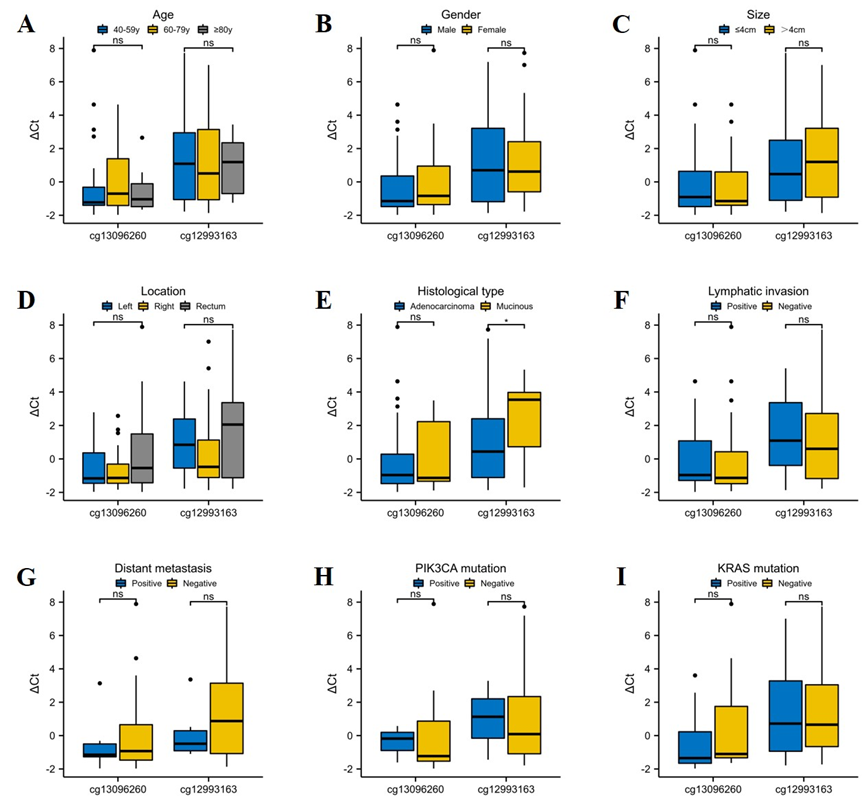
**

**Additional file 1: Figure S2**. Relationship between methylation levels of cg13096260 and cg12993163, and clinicopathological characteristics of tissue samples. a–i show age, sex, tumor size, tumor location, histological type, lymphatic invasion, distant metastasis, PIK3CA mutation, and KRAS mutation.

**
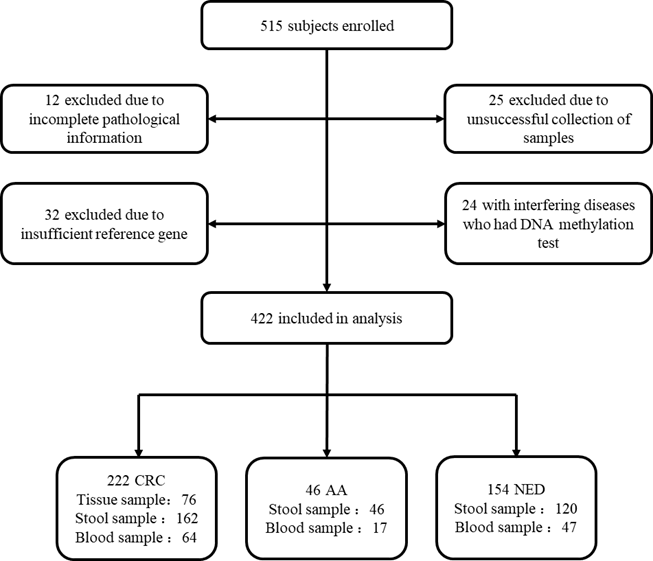
**

**Additional file 1: Figure S3**. Flowchart of sample collection.
